# Analysis of seven SARS-CoV-2 rapid antigen tests in detecting omicron (B.1.1.529) versus delta (B.1.617.2) using cell culture supernatants and clinical specimens

**DOI:** 10.1007/s15010-022-01844-5

**Published:** 2022-05-20

**Authors:** Sabrina Jungnick, Bernhard Hobmaier, Natali Paravinja, Lena Mautner, Mona Hoyos, Regina Konrad, Maren Haase, Armin Baiker, Ute Eberle, Magdalena Bichler, Bianca Treis, Mercy Okeyo, Barbara Streibl, Clara Wimmer, Sabrina Hepner, Annika Sprenger, Carola Berger, Laura Weise, Alexandra Dangel, Siegfried Ippisch, Walter Jonas, Manfred Wildner, Bernhard Liebl, Nikolaus Ackermann, Andreas Sing, Volker Fingerle, the Bavarian SARS-CoV-2-Public Health Laboratory Team, Vadim Balakin, Bernadett Bartha-Dima, Katja Bengs, Anja Berger, Kerstin Boll, Anja Carl, Jürgen Christian, Juliana Drdlicek, David Eisenberger, Jennifer Flechsler, Lars Gerdes, George Githure, Janani Govindaswamy, Christine Hupfer, Johannes Lutmayr, Gabriele Margos, Roswitha Müller, Silke Nickel, Melanie Pavlovic, Sven Pecoraro, Daniel Reichwald, Robert Ethan Rollins, Isabel Sahm, Melanie Schauer, Sandra Schmidt, Gesine Schulze, Anika Schülein, Eva-Maria Schürmann, Nelly Scuda, Judith Seebach, Stefanie Singer, Thorsten Stellberger, Christian Tuschak, Pia Zimmermann

**Affiliations:** 1 Public Health Microbiology Unit, Bavarian Health and Food Safety Authority, Oberschleißheim, Germany; 2 Unit of Molecular Biologic Analytics and Biogenetics, Bavarian Health and Food Safety Authority, Oberschleißheim, Germany; 3 Ludwig Maximilian University, Munich, Germany; 4 Bavarian State Institute of Health, Oberschleißheim, Germany; 5 Bavarian Pandemic Warehouse, Bavarian Health and Food Safety Authority, Oberschleißheim, Germany

**Keywords:** SARS-CoV-2 rapid antigen test, Comparison, Omicron (B.1.1.529), Delta (B.1.617.2), Variants of concern

## Abstract

**Purpose:**

Omicron is rapidly spreading as a new SARS-CoV-2 variant of concern (VOC). The question whether this new variant has an impact on SARS-CoV-2 rapid antigen test (RAT) performance is of utmost importance. To obtain an initial estimate regarding differences of RATs in detecting omicron and delta, seven commonly used SARS-CoV-2 RATs from different manufacturers were analysed using cell culture supernatants and clinical specimens.

**Methods:**

For this purpose, cell culture-expanded omicron and delta preparations were serially diluted in Dulbecco’s modified Eagle’s Medium (DMEM) and the Limit of Detection (LoD) for both VOCs was determined. Additionally, clinical specimens stored in viral transport media or saline (*n* = 51) were investigated to complement in vitro results with cell culture supernatants. Ct values and RNA concentrations were determined via quantitative reverse transcription polymerase chain reaction (RT-qPCR).

**Results:**

The in vitro determination of the LoD showed no obvious differences in detection of omicron and delta for the RATs examined. The LoD in this study was at a dilution level of 1:1,000 (corresponding to 3.0—5.6 × 10^6^ RNA copies/mL) for tests I–V and at a dilution level of 1:100 (corresponding to 3.7—4.9 × 10^7^ RNA copies/mL) for tests VI and VII. Based on clinical specimens, no obvious differences were observed between RAT positivity rates when comparing omicron to delta in this study setting. Overall positivity rates varied between manufacturers with 30–81% for omicron and 42–71% for delta. Test VII was only conducted in vitro with cell culture supernatants for feasibility reasons. In the range of Ct < 23, positivity rates were 50–100% for omicron and 67–93% for delta.

**Conclusion:**

In this study, RATs from various manufacturers were investigated, which displayed no obvious differences in terms of analytical LoD in vitro and RAT positivity rates based on clinical samples comparing the VOCs omicron and delta. However, differences between tests produced by various manufacturers were detected. In terms of clinical samples, a focus of this study was on specimens with high virus concentrations. Further systematic, clinical and laboratory studies utilizing large datasets are urgently needed to confirm reliable performance in terms of sensitivity and specificity for all individual RATs and SARS-CoV-2 variants.

**Supplementary Information:**

The online version contains supplementary material available at 10.1007/s15010-022-01844-5.

## Introduction

The new SARS-CoV-2 variant of concern (VOC) omicron, first described publicly on November 24th 2021 in South Africa, is spreading rapidly throughout the globe, overtaking delta as the dominant variant in many countries [1, 2]. Thus, the question whether this new variant has an impact on diagnostic equipment performance is of utmost importance. Besides multiple mutations in the spike (S)-protein, omicron BA.1 shows three single characteristic point mutations and one deletion in the nucleocapsid protein (N-protein) (P13L, Δ31-33, R203K, G204R) [3]. Since the N-protein is the target protein of most SARS-CoV-2 rapid antigen tests (RATs), it is crucial to investigate whether the corresponding mutations affect RAT performance [4].

To obtain a basic understanding of the performance of RATs in detecting omicron, RATs for professional use and self-application from seven widely used manufacturers (Table 1) listed by the German Federal Institute for Drugs and Medicinal Devices ‘BfArM’ (except for test VII) were tested with in vitro cell culture supernatants (test I to VII) and clinical specimens (test I to VI). All deployed tests target the SARS-CoV-2 N-protein and are lateral flow immunoassays [5]. Tests I–VI are colourimetric, whereas test VII is fluorescence based and requires the manufacturer’s specific analyser for interpretation of results.

**Table 1:** RATs investigated in this study

Test	Name	Manufacturer
Test I	SARS-CoV-2 Rapid Antigen Test (self-test^1^) *	Roche, Mannheim, Germany
Test II	CLINITEST Rapid COVID-19 Antigen Self-Test *	Siemens Healthineers, Erlangen, Germany
Test III	Rapid SARS-CoV-2 Antigen Test Card *	Xiamen Boson Biotech Co., Xiamen, China
Test IV	Panbio COVID-19 Ag RAPID TEST DEVICE (NASAL)	Abbott, Jena, Germany
Test V	NADAL COVID-19 Ag Test	Nal von Minden, Moers, Germany
Test VI	BIOCREDIT COVID-19 Ag One Step Rapid Test	Rapigen Inc., Anyang-si, South Korea
Test VII^2^	Sofia SARS Antigen FIA	Quidel Corporation, San Diego, CA, USA

* Officially approved for self-testing according to German approval regulations. ^1^ Evaluation with cell culture supernatants according to the manufacturer’s instructions for self-testing, evaluation with clinical samples according to the manufacturer’s instructions for professional use testing. ^2^ Evaluation only with cell culture supernatants

## Laboratory performance of RATs with cell culture-derived omicron and delta

RAT performance was evaluated in vitro by determining analytical Limits of Detection (LoDs) in DMEM (Dulbecco’s modified Eagle’s Medium) with infectious SARS-CoV-2 omicron (B.1.1.529, BA.1) and delta (B.1.617.2, AY.122) samples (confirmed by Whole Genome Sequencing (WGS)) derived from cell culture in Vero E6 cells (ATCC CRL-1586, RRID: CVCL_0574) as described [6]. Viral load was determined by quantitative real-time PCR using the *EDX SARS-CoV-2 Positive Run Control* (Exact Diagnostics, Fort Worth, TX USA) as calibrator. The LoD was determined as the dilution level at which all three replicates were considered positive by two or, in case of ambiguity, three investigators, independently and blinded regarding dilution levels. For test VII, a sample analyser from the manufacturer was used for result interpretation.

Based on the analytical LoD of RATs evaluated, no distinct performance differences between omicron and delta were determined in this investigated setting (Table 2). Five tests were able to detect both virus variants at least up to a dilution of 1:1,000 (corresponding to 3.0—5.6 × 10^6^ RNA copies/mL), while tests VI and VII displayed positive results only up to a dilution level of 1:100 (corresponding to 3.7—4.9 × 10^7^ RNA copies/mL) (Table 2). Some tests showed positive results at higher dilution levels for either omicron or both VOCs under study but in less than three replicates. It is important to mention here that there were small differences in concentrations between the omicron and delta dilutions, with omicron containing slightly higher genome copy numbers (Table 2).

**Table 2:**
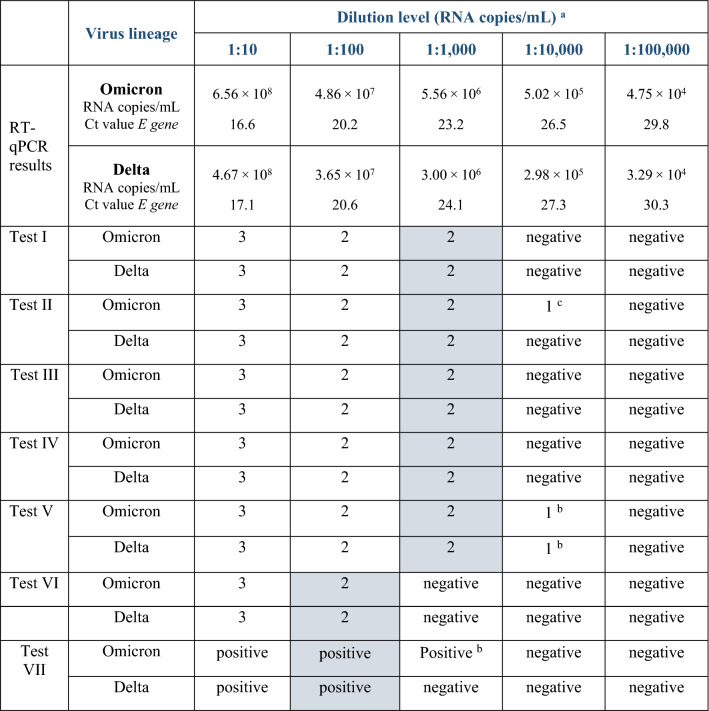
Limits of detection with cell culture supernatants from omicron and delta based on virus dilution series in DMEM

The test results are described as negative (no target band visible) or as various degrees of positive, depending on the strength of visibility of the test band (except test VII, due to platform-based result interpretation as required by the manufacturer): (1) very faint, (2) weaker than control band and (3) equally strong or stronger than control band. LoD was determined as the dilution level at which all three replicates were considered positive by at least two investigators independently and blinded regarding dilution levels

^a^Cells shaded in grey-blue colour indicate the dilution determining the LoD

^b^Only two of three replicates showed a very faint target band

^c^Only one out of three replicates showed a very faint band. Ct: cycle threshold; *E* gene: envelope protein gene; Infectivity of initial preparations (TCID50/mL) omicron: 3.16 × 10^4^, delta: 2.11 × 10^5^. Displayed RNA concentrations and Ct values are mean values of duplicates

## Investigating SARS-CoV-2 RATs with clinical specimens containing omicron or delta

Additionally, RAT performance was analysed utilizing clinical specimens positive for omicron or delta to complete in vitro results with cell culture supernatants. Clinical specimens (*n* = 51; nasopharyngeal/oropharyngeal swabs in viral transport media (VTM) or saline (NaCl)) were collected between the 13^th^ and 24^th^ of December 2021 at the Bavarian Health and Food Safety Authority as remnants of routine diagnostics and stored at 2–8 °C (Supplementary Table). Lineages were confirmed by variant-specific RT-qPCR (ViRSNiP assay, TIB MOLBIOL, Berlin, Germany) and WGS (except no. 24: due to low virus concentration confirmation only by variant-specific RT-qPCR). All clinical omicron samples were B.1.1.529, BA.1, while delta samples varied in subtype. To minimize the influence of confounding factors (e.g. medium, sampling date) and to generate comparable groups of samples containing the variants under study, panels with an approximately equal distribution of I) Cycle threshold (Ct) values, II) transport media and III) sampling dates were assembled. Even so, minor differences between panels remained present (Supplementary Table). Only viral transport media without guanidinium were included because of its protein-denaturizing effects. To obtain comparable Ct values within this study, all clinical samples were quantified in one collective RT-qPCR run prior to RAT testing as described previously (*AmpliCube Coronavirus SARS-CoV-2 RT-qPCR Kit* (Mikrogen, Neuried, Germany) on a *BioRad CFX96 Real-Time RT-qPCR Detection System* (BioRad, Feldkirchen, Germany), extraction on a *Microlab STAR* (Hamilton, Reno, NV, USA), using the *RNAdvance Viral Large GRP Kit* (Beckmann-Coulter Life Sciences, Nyon, Switzerland)) [6, 7]. A majority of clinical samples utilized in this study contained high viral loads.

For reasons of standardization and comparability, the methodological procedure was largely based on the protocol developed by the Paul-Ehrlich-Institute but without prior pooling of samples [5]. In short, 50 µL of each sample was inoculated into the respective RAT buffers by pipetting. Sample media were either Virocult (Medical Wire and Equipment Virocult, Wiltshire, UK) or saline. RT-qPCR-negative patient samples in Virocult and saline were included as negative controls for all RATs evaluated. Further procedural steps, such as number of drops or incubation time, were carried out strictly according to the manufacturer’s instructions of the respective RATs in a BSL-3 laboratory. Samples were considered positive if two investigators could still clearly visually identify positive test bands in duplicates independently and blinded regarding concentration levels. In case of ambiguity, a third investigator was consulted.

## Statistical analysis of results with clinical specimens

Delta and omicron samples were tested for differences of their Ct values in respect to their variance (*F*-test, two sided) and mean (*t*-test, two sided, equal variance). No differences were found at a statistical significance level (*p*-values ≥ 0.05). Based on these data, three hypotheses were tested in an exploratory approach (null hypotheses) at a statistical significance level of *p* < 0.05 (total samples without stratification for Ct values; using Fisher’s exact test and Chi-square test for homogeneity):

1. Test results of sensitivity towards omicron do not differ from test results for delta (Fisher’s exact test).
2. Tests do not perform significantly different in respect to sensitivity towards delta (Chi-square test for homogeneity).
3. Tests do not perform significantly different in respect to sensitivity towards omicron (Chi-square test for homogeneity).

Hypothesis testing was performed using statistical tools available online [8, 9].

## SARS-CoV-2 RAT results with clinical specimens of omicron and delta

In this study setting, the investigation of RATs with clinical samples showed no distinct differences between positivity rates of omicron versus delta, which is in line with in vitro laboratory analysis using cell culture supernatants. For Ct > 23, the sample panel was too small and partially unbalanced in terms of viral loads between omicron and delta, so that no distinct conclusions based on clinical specimens could be drawn for Ct > 23.

A total of 27 omicron and 24 delta clinical samples were examined. Depending on the manufacturer, 30–81% of all omicron samples and 42–71% of all delta samples were detected as positive (Table 3). There were no clear performance differences between the VOCs omicron and delta for high viral loads (Ct < 23; mean Ct 20.3 (omicron) and 19.4 (delta)) in five out of six tests (positivity rate: 80–100%). Test VI showed lower positivity rates with 50% for omicron and 67% for delta at Ct < 23. For Ct values 23–25.5, positivity rates varied strongly (0–100%) between different RATs with higher rates for omicron. For Ct > 25.5, only one positive sample was detected in one of the RATs (test V, omicron). Test V showed very faint test bands in duplicates in a negative saline control (Supplementary Table).

**Table 3:** Positivity rates of RATs with clinical samples of omicron and delta

Test	Total samples detected		Ct < 23*		Ct 23—25.5 **		Ct > 25.5 **	
	Omicron	Delta	Omicron	Delta	Omicron	Delta	Omicron	Delta
Test I	67% (18/27)	63% (15/24)	88% (14/16)	93% (14/15)	67% (4/6)	20% (1/5)	0% (0/5)	0% (0/4)
Test II	67% (18/27)	58% (14/24)	81% (13/16)	93% (14/15)	83% (5/6)	0% (0/5)	0% (0/5)	0% (0/4)
Test III	81% (22/27)	71% (17/24)	100% (16/16)	93% (14/15)	100% (6/6)	60% (3/5)	0% (0/5)	0% (0/4)
Test IV	63% (17/27)	58% (14/24)	81% (13/16)	87% (13/15)	67% (4/6)	20% (1/5)	0% (0/5)	0% (0/4)
Test V	63% (17/27)	50% (12/24)	81% (13/16)	80% (12/15)	50% (3/6)	0% (0/5)	20% (1/5)	0% (0/4)
Test VI	30% (8/27)	42% (10/24)	50% (8/16)	67% (10/15)	0% (0/6)	0% (0/5)	0% (0/5)	0% (0/4)

*Mean Ct value: 20.3 (omicron) and 19.4 (delta) **CAVE: slightly uneven distribution of Ct values, see Supplementary Table. Tests conducted on two evaluation days (day one: test III, day two: other tests)

The null hypotheses 1 and 2 could not be rejected. Null hypothesis 3 was rejected at a *p*-level < 0.05, indicating inhomogeneity of test performance among the six tests under investigation in respect to omicron. The Chi-square test for homogeneity was repeated in respect to the sensitivity of this result towards the performance of test VI and regarding the possible influence of high Ct values. After exclusion of test VI, the null hypothesis could not be rejected anymore (*p* = 0.569). This finding remained stable when also excluding samples with Ct values > 23 (*p* = 0.465).

## Discussion

Clinical and laboratory data evaluating the effects of the new VOC omicron on RAT performance are crucial but scarce. We investigated seven widely used SARS-CoV-2 RATs and found no distinct differences in analytical LoDs and positivity rates with clinical samples comparing the two VOCs omicron and delta, whereas test VII was only tested for analytical LoD. However, there were partial differences in results between various manufacturers. Differences in the general sensitivity of tests were also reported by the German Paul-Ehrlich-Institute and the Robert-Koch-Institute who tested 122 CE-certified RATs systematically with 26 RATs missing the sensitivity criteria [5]. In this study, results of the laboratory investigations using cell culture supernatants showed no clear differences in detecting omicron compared to delta. This result is in line with results of investigations utilizing clinical samples at high viral loads. Slightly lower positivity rates for omicron compared to delta with clinical samples for test VI were not observed in results with cell culture supernatants. In general, this study utilizes analytical laboratory data. Data based on clinical specimens were only considered in a descriptive way due to small clinical sample size and partly varying Ct value distributions in samples positive for omicron or delta. Nevertheless, among the six RATs tested with clinical specimens, exploratory hypothesis testing indicated a statistically significant heterogeneity in test performance regarding test sensitivity towards omicron (*p* < 0.05) between RATs of different manufacturers. This heterogeneity could be linked to the poorer performance of one of the six tests under investigation. Within these six tests under investigation, no significant difference in respect to test sensitivity towards omicron as compared to test sensitivity towards delta was detected.

Results of this study agree with those of Deerain et al. (2021), who described no distinct performance differences between omicron and delta for ten evaluated RATs using cultured virus dilutions, which included two RATs (test I and test IV) also present in our study [10]. Similarly, Regan et al. (2021) reported comparable performances between omicron and delta, particularly at high virus concentrations, for the Abbott BinaxNow rapid antigen test using clinical samples [11]. Other studies, however, described lower sensitivity of tests evaluated with omicron compared to delta [12, 13]. In comparison to RATs investigated in our study, Bekliz et al. (2021) showed lower sensitivities with omicron compared to delta for test IV with clinical specimens and cell culture-expanded samples [12]. Osterman et al. (2022) showed lower sensitivities for omicron in test III with clinical samples, while results with cell culture-expanded samples were reversed [13].

It is important to mention some limitations of the current study, which could influence interpretation of the results. The experimental set-up with tenfold dilution steps may mask differences in LoDs smaller than the tested tenfold increments. Additionally, slight variations in the concentrations of omicron and delta preparations should be considered, as this fact could influence the results of the current study. In previous studies, DMEM was considered a suitable medium for investigations of cell culture samples [6]. As only few RATs are approved by the manufacturer for testing from specific VTMs and for purposes of consistency, a sample volume of 50 µL was elected. This, however, may not exactly reflect the suggested volumes of certain manufacturers and was chosen for comparability among the tests and to other studies [5, 6, 11, 13]. Moreover, there could be dilution effects introduced due to the variable buffer volumes provided by the RAT manufacturers and, additionally, the differences in sample loaded onto the test cassette according to the respective manufacturer’s instructions. Test band intensities of tests I–VI were quantified by visual inspection instead of software-guided image analysis, which does reflect the conditions present in the clinical and practical application of these tests but may lead to investigator-dependent variations in the perceived signal strength. An exemption is test VII, which was interpreted by a test-specific detection device as required by the manufacturer. RAT investigations with clinical specimens were conducted on two different test days. As residual samples from routine diagnostics were used, some samples exceeded the storage time specified by the manufacturers. Respective samples were submitted from different external sources and were processed on different days as part of routine diagnostics. Uniform conditions of sample storage (i.e. cold chain) on the part of the submitting institution or during transport can therefore not be guaranteed. A great benefit of this study is that examinations were not only conducted with cell culture supernatants*,* but also with clinical samples. In addition, the methodological approach was largely based on those of large studies, such as the Paul-Ehrlich-Institute and on earlier publications of the working group concerning the analytical LoD of RATs with other VOCs [5, 6]. A standardized procedure was established for RAT investigation with replicable virus and clinical samples of routine diagnostics, and all tests were examined on one, or in the case of clinical samples, a maximum of two consecutive test days.

Overall, the RATs examined detected omicron comparably to delta in this study. Analytical LoD and positivity rates were determined using cell culture-expanded virus and clinical samples with a focus on high virus concentrations. However, differences in LoD and positivity rates were found between the different manufacturers. Whether the general performance of RATs from different manufacturers is varying systematically in a relevant magnitude is subject to further research, taking the limited study size and possible chance effects into account. Besides sensitivity, specificity of RATs plays a decisive role within RAT evaluation. Corresponding systematic clinical and laboratory studies with large sample sizes for all individual tests and variants are urgently needed to confirm reliable RAT performance.

## Supplementary Information

Below is the link to the electronic supplementary material.

Supplementary file1 (PDF 296 kb)

## References

[1] World Health Organization. Classification of Omicron (B.1.1.529): SARS-CoV-2 Variant of Concern. 2021. https://www.who.int/news/item/26-11-2021-classification-of-omicron-(b.1.1.529)-sars-cov-2-variant-of-concern Accessed 20 Jan 2022.

[2] Viana R, Moyo S, Amoako DG, Tegally H, Scheepers C, Althaus CL (2022). Rapid epidemic expansion of the SARS-CoV-2 Omicron variant in southern Africa. Nature.

[3] Latif AA, Mullen JL, Alkuzweny M, Tsueng G, Cano M, Haag E, et al. B.1.1.529 Lineage Report. outbreak.info. 2022. https://outbreak.info/situation-reports?pango=B.1.1.529 Accessed 20 Jan 2022.

[4] Ferré VM, Peiffer-Smadja N, Visseaux B, Descamps D, Ghosn J, Charpentier C (2021). Omicron SARS-CoV-2 variant: What we know and what we don't. Anaesthesia Crit Care Pain Med..

[5] Scheiblauer H, Filomena A, Nitsche A, Puyskens A, Corman VM, Drosten C, et al. Comparative sensitivity evaluation for 122 CE-marked rapid diagnostic tests for SARS-CoV-2 antigen, Germany, September 2020 to April 2021. Eurosurveillance. 2021; 26(44). 10.2807/1560-7917.es.2021.26.44.2100441 10.2807/1560-7917.ES.2021.26.44.2100441 PMC8569926 34738515

[6] Jungnick S, Hobmaier B, Mautner L, Hoyos M, Haase M, Baiker A, et al. In Vitro Rapid Antigen Test Performance with the SARS-CoV-2 Variants of Concern B.1.1.7 (Alpha), B.1.351 (Beta), P.1 (Gamma), and B.1.617.2 (Delta). Microorganisms. 2021; 9(9): 1967. 10.3390/microorganisms9091967 10.3390/microorganisms9091967 PMC8465346 34576862

[7] Eberle U, Wimmer C, Huber I, Neubauer-Juric A, Valenza G, Ackermann N (2021). Comparison of nine different commercially available molecular assays for detection of SARS-CoV-2 RNA. Eur J Clin Microbiol Infect Dis.

[8] Preacher KJ and Briggs NE. Calculation for Fisher's Exact Test: An interactive calculation tool for Fisher's exact probability test for 2 x 2 tables [Computer software]. 2001. http://quantpsy.org. Accessed 19 Apr 2022.

[9] Preacher KJ. Calculation for the chi-square test: An interactive calculation tool for chi-square tests of goodness of fit and independence [Computer software]. 2001. http://quantpsy.org. Accessed 19 Apr 2022.

[10] Deerain J, Druce J, Tran T, Batty M, Yoga Y, Fennell M (2021). Assessment of the analytical sensitivity of ten lateral flow devices against the SARS-CoV-2 omicron variant. J Clin Microbiol.

[11] Regan J, Flynn JP, Choudhary MC, Uddin R, Lemieux J, Boucau J, et al. Detection of the Omicron Variant Virus With the Abbott BinaxNow SARS-CoV-2 Rapid Antigen Assay. Open Forum Infectious Diseases. 2022; 9(3). 10.1093/ofid/ofac022 10.1093/ofid/ofac022 PMC8842316 35169591

[12] Bekliz M, Perez-Rodriguez F, Pulhach O, Adea K, Melanica SM, Baggio S, et al. Sensitivity of seven SARS-CoV-2 antigen-detecting rapid tests for Omicron variant. medRxiv. 2021; 10.1101/2021.12.18.21268018. Accessed 16 Mar 2022.

[13] Osterman A, Badell I, Basara E, Stern M, Kriesel F, Eletreby M (2022). Impaired detection of omicron by SARS-CoV-2 rapid antigen tests. Med Microbiol Immunol.

