# Supplementary material for: Analysis of seven SARS-CoV-2 rapid antigen tests in detecting omicron (B.1.1.529) versus delta (B.1.617.2) using cell culture supernatants and clinical specimens
Source: Infection. 2022 May 20;51(1):239–45. doi: 10.1007/s15010-022-01844-5 (PMC9122478; doi:10.1007/s15010-022-01844-5)
Supplement: Supplementary file 1 — Supplementary file1 (PDF 296 kb) [file 15010_2022_1844_MOESM1_ESM.pdf]

Infection

Analysis of seven SARS-CoV-2 rapid antigen tests in detecting omicron (B.1.1.529) versus delta (B.1.617.2) using cell culture supernatants and clinical specimens

Sabrina Jungnick[1,2], Bernhard Hobmaier[1,2], Natali Paravinja[1,2], Lena Mautner[3], Mona Hoyos[3], Regina Konrad[2], Maren Haase[3], Armin Baiker[3], Ute Eberle[2], Magdalena Bichler[2], Bianca Treis[2], Mercy Okeyo[2], Barbara Streibl[2], Clara Wimmer[2], Sabrina Hepner[2], Annika Sprenger[2], Carola Berger[2], Laura Weise[2], Alexandra Dangel[2], Siegfried Ippisch[6], Walter Jonas[5], Manfred Wildner[4,5], Bernhard Liebl[4,5], Nikolaus Ackermann[2], Andreas Sing[2,4], Volker Fingerle[2], the Bavarian SARS-CoV-2-Public Health Laboratory Team[2,7]

1. These authors contributed equally to this work
2. Public Health Microbiology Unit, Bavarian Health and Food Safety Authority, Oberschleißheim, Germany
3. Unit of molecular biologic analytics and biogenetics, Bavarian Health and Food Safety Authority, Oberschleißheim, Germany
4. Ludwig Maximilian University, Munich, Germany
5. Bavarian State Institute of Health, Oberschleißheim, Germany
6. Bavarian Pandemic Warehouse, Bavarian Health and Food Safety Authority, Oberschleißheim, Germany
7. Members of the Bavarian SARS-CoV-2-Public Health Laboratory Team are listed in acknowledgement

Correspondence: Volker Fingerle (volker.fingerle@lgl.bayern.de)

SUPPLEMENTARY INFORMATION

SUPPLEMENTARY TABLE Individual results and background information of clinical omicron and delta samples

Test results are described as negative (0) or as various degrees of positive, depending on the strength of visibility of the test band: (1) very faint, (2) weaker than control band, (3) equally strong or stronger than control band. * confirmed bei WGS, except no. 24 (due to low virus concentration WGS not successful, confirmed by variant specific RT-qPCR). N/A not available. Negative controls no. 52-54.

	Result (replicate 1/ replicate 2)						Background Information			
Sample number	Test I	Test II	Test III	Test IV	Test V	Test VI	E-gene Ct value	Medium	Sampling date	Variant of Concern*
1	3/3	3/3	3/3	3/3	2/2	2/2	15.38	Virocult	21.12.2021	delta
2	2/2	2/2	3/3	2/2	2/2	2/2	16.87	NaCl	24.12.2021	delta
3	3/3	3/3	3/3	3/3	2/2	2/2	17.14	NaCl	20.12.2021	delta
4	2/2	2/2	3/3	2/2	2/2	2/2	17.80	Virocult	22.12.2021	delta
5	3/3	3/3	3/3	2/2	0/0	2/2	18.80	NaCl	22.12.2021	delta
6	2/2	2/2	2/2	2/2	2/1	1/0	18.95	NaCl	22.12.2021	delta
7	2/2	2/2	2/2	0/0	2/2	1/1	19.01	Virocult	22.12.2021	delta
8	2/2	2/2	3/3	2/2	2/2	2/2	19.57	NaCl	22.12.2021	delta
9	2/2	2/2	2/2	2/2	1/1	0/0	19.96	Virocult	23.12.2021	delta
10	1/1	2/2	2/2	2/2	1/1	0/0	20.27	Virocult	22.12.2021	delta
11	2/2	3/3	3/3	2/2	3/3	2/2	20.41	Virocult	22.12.2021	delta
12	2/2	2/2	2/2	2/2	1/0	2/2	21.07	Virocult	13.12.2021	delta
13	2/2	2/2	2/2	2/2	1/1	2/2	21.68	NaCl	17.12.2021	delta
14	0/0	0/0	0/0	0/0	1/0	0/0	21.88	Virocult	13.12.2021	delta
15	2/2	2/2	2/2	1/1	1/1	0/0	22.08	Virocult	17.12.2021	delta
16	0/0	0/0	1/1	0/0	0/0	0/0	23.22	Virocult	14.12.2021	delta
17	0/0	0/0	0/0	0/0	0/0	0/0	23.73	Virocult	20.12.2021	delta
18	0/0	0/0	1/1	0/0	0/0	0/0	24.20	Virocult	16.12.2021	delta
19	0/0	0/0	0/0	0/0	0/0	0/0	24.52	Virocult	22.12.2021	delta
20	1/1	0/0	1/1	2/2	0/0	0/0	25.20	NaCl	20.12.2021	delta
21	0/0	0/0	0/0	0/0	0/0	0/0	26.00	Virocult	17.12.2021	delta
22	0/0	0/0	0/0	0/0	0/0	0/0	28.01	Virocult	17.12.2021	delta
23	0/0	0/0	0/0	0/0	0/0	0/0	29.17	Virocult	23.12.2021	delta
24	0/0	0/0	0/0	0/0	0/0	0/0	35.53	Virocult	21.12.2021	delta
25	3/3	3/3	3/3	3/3	3/3	3/3	15.50	Virocult	22.12.2021	omicron
26	3/3	3/3	3/3	3/3	2/2	2/2	18.00	NaCl	23.12.2021	omicron
27	3/3	2/2	3/3	3/3	2/2	2/2	18.58	Virocult	21.12.2021	omicron
28	2/2	2/2	3/3	2/2	2/2	2/2	18.96	Virocult	21.12.2021	omicron
29	2/2	2/2	2/2	2/2	2/2	0/1	19.48	Virocult	23.12.2021	omicron
30	2/2	0/0	3/3	2/2	2/2	1/1	19.59	NaCl	23.12.2021	omicron
31	1/1	0/0	2/2	0/0	0/0	0/0	20.14	Virocult	23.12.2021	omicron
32	2/2	2/2	2/2	2/2	2/2	2/2	20.86	NaCl	23.12.2021	omicron
33	2/2	2/2	3/3	2/2	2/2	2/2	21.09	Virocult	16.12.2021	omicron
34	2/2	2/2	2/2	2/2	1/1	0/0	21.21	NaCl	23.12.2021	omicron

35	2/2	2/2	2/2	2/2	1/1	0/1	21.30	Virocult	16.12.2021	omicron
36	1/1	2/2	2/2	2/2	0/0	0/0	21.45	Virocult	16.12.2021	omicron
37	0/0	0/0	1/1	0/0	1/1	0/0	22.09	Virocult	20.12.2021	omicron
38	1/1	2/2	2/2	1/1	2/2	0/0	22.19	NaCl	23.12.2021	omicron
39	1/0	1/1	2/2	0/0	2/2	0/0	22.38	Virocult	20.12.2021	omicron
40	2/2	2/2	2/2	2/2	2/0	1/1	22.45	NaCl	14.12.2021	omicron
41	1/1	2/2	2/2	2/2	2/2	0/0	23.09	Virocult	16.12.2021	omicron
42	1/1	0/0	2/2	2/2	0/0	0/0	23.15	Virocult	20.12.2021	omicron
43	2/2	2/2	2/2	1/1	2/2	1/0	23.48	Virocult	21.12.2021	omicron
44	0/0	1/1	2/2	0/0	0/0	0/0	23.56	Virocult	22.12.2021	omicron
45	0/0	1/1	1/1	0/0	0/0	0/0	23.83	Virocult	13.12.2021	omicron
46	2/2	1/1	2/2	2/2	1/1	0/0	24.33	Virocult	16.12.2021	omicron
47	0/0	0/0	0/0	0/0	2/2	0/0	26.42	NaCl	23.12.2021	omicron
48	0/0	0/0	0/0	0/0	0/0	0/0	26.86	Virocult	20.12.2021	omicron
49	0/0	0/0	0/0	0/0	0/0	0/0	27.99	Virocult	14.12.2021	omicron
50	0/0	0/0	0/0	0/0	0/0	0/0	28.07	Virocult	20.12.2021	omicron
51	0/0	0/0	0/0	0/0	0/0	0/0	31.36	Virocult	22.12.2021	omicron
52	0/0	0/0	0/0	0/0	0/0	0/0	N/A	Virocult	27.12.2021	negative
53	0/0	0/0	0/0	0/0	1/1	0/0	N/A	NaCl	13.12.2021	negative
54	0/0	0/0	0/0	0/0	0/0	0/0	N/A	NaCl	13.12.2021	negative
